# Supplementary material for: Overall water splitting by graphdiyne-exfoliated and -sandwiched layered double-hydroxide nanosheet arrays
Source: Nat Commun. 2018 Dec 14;9:5309. doi: 10.1038/s41467-018-07790-x (PMC6294247; doi:10.1038/s41467-018-07790-x)
Supplement: Supplementary file 1 — Supplementary Information [file 41467_2018_7790_MOESM1_ESM.pdf]

Supplementary Information

Overall water splitting by graphdiyne-exfoliated and -sandwiched layered double-hydroxide nanosheet arrays

Lan Hui[1,3], Yurui Xue[1,2*], Bolong Huang[5], Huidi Yu[1], Chao Zhang[1], Danyan Zhang[1], Dianzeng Jia[3*], Yingjie Zhao[2], Yongjun Li[1], Huibiao Liu[1] and Yuliang Li[1,2,4*]

[1]Key Laboratory of Organic Solids, Institute of Chemistry, Chinese Academy of Sciences, Beijing 100190, PR China.

[2]School of Polymer Science and Engineering, Qingdao University of Science and Technology, Qingdao 266042, P.R. China.

[3]Key Laboratory of Energy Materials Chemistry, Ministry of Education; Key laboratory of Advanced Functional Materials, Autonomous Region; Institute of Applied Chemistry, Xinjiang University, Urumqi 830046, Xinjiang, P. R. China.

[4]University of Chinese Academy of Sciences, Beijing 100049, PR China.

[5]Department of Applied Biology and Chemical Technology, The Hong Kong Polytechnic University, Hung Hom, Kowloon, Hong Kong SAR, China.

*e-mail: xueyurui@iccas.ac.cn, jdz@xju.edu.cn, ylli@iccas.ac.cn

Supplementary Figures

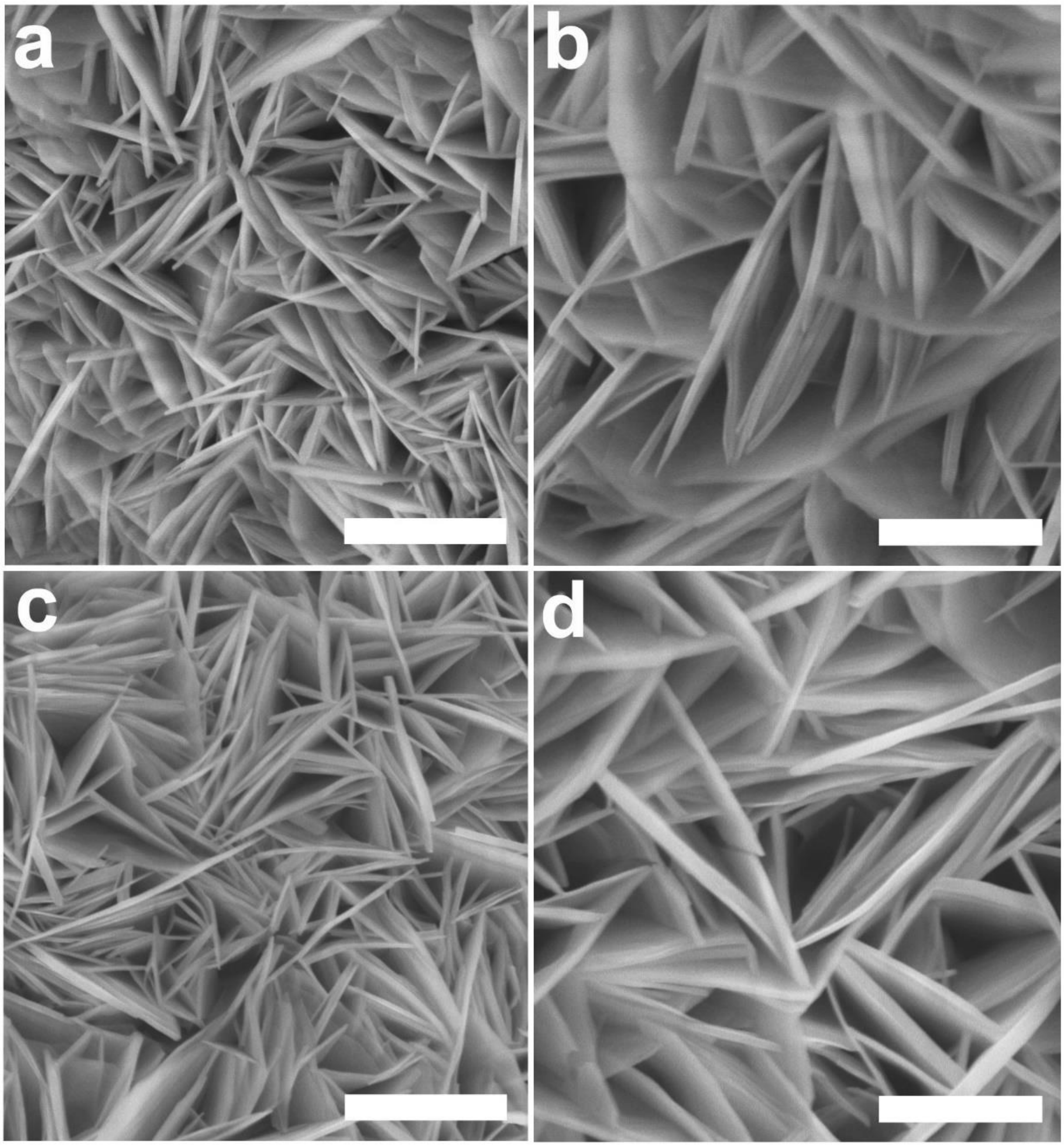

Supplementary Figure 1. Morphology characterizations

SEM images of ICLDH/NF **a, b** before and **c, d** after being treated by pyridine. Scale bars: a and c, 1 μm; b and d, 500 nm.

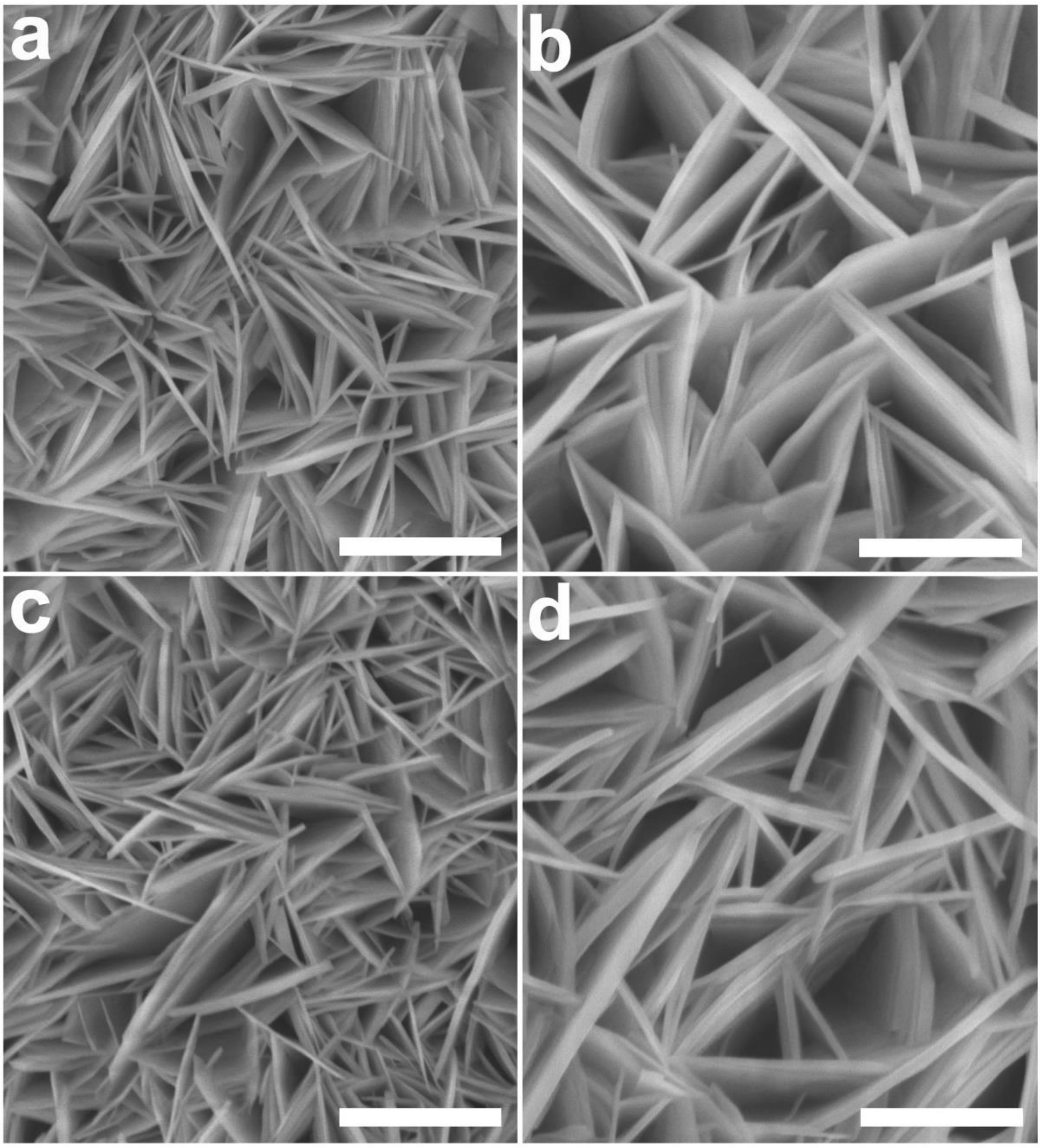

Supplementary Figure 2. Morphology characterizations

SEM images of ICLDH/NF **a, b** before and **c, d** after being treated by TMEDA. Scale bars: a and c, 1 μm; b and d, 500 nm.

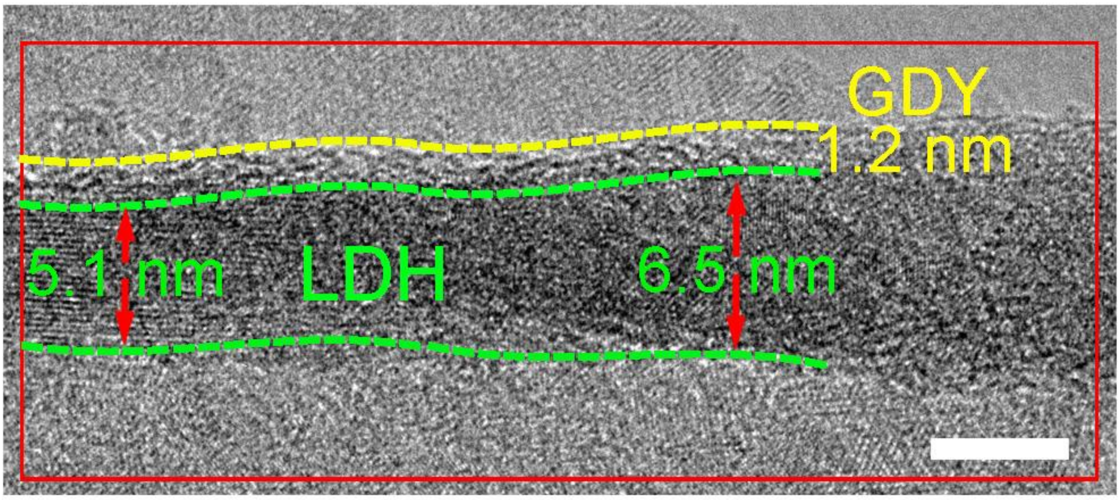

Supplementary Figure 3. HRTEM image of the lateral standing e-ICLDH@GDY nanosheet

Because of the imaging angle, the GDY layer coated on the other side was not observed. The thickness of GDY (or LDH) is estimated from the number of fringes corresponding to the distance between two consecutive carbon (or LDH) fringes. Therefore, the thickness of GDY and LDH are determined to be about 1.2 nm and 5.1~6.5 nm, respectively. Scale bars: 5 nm.

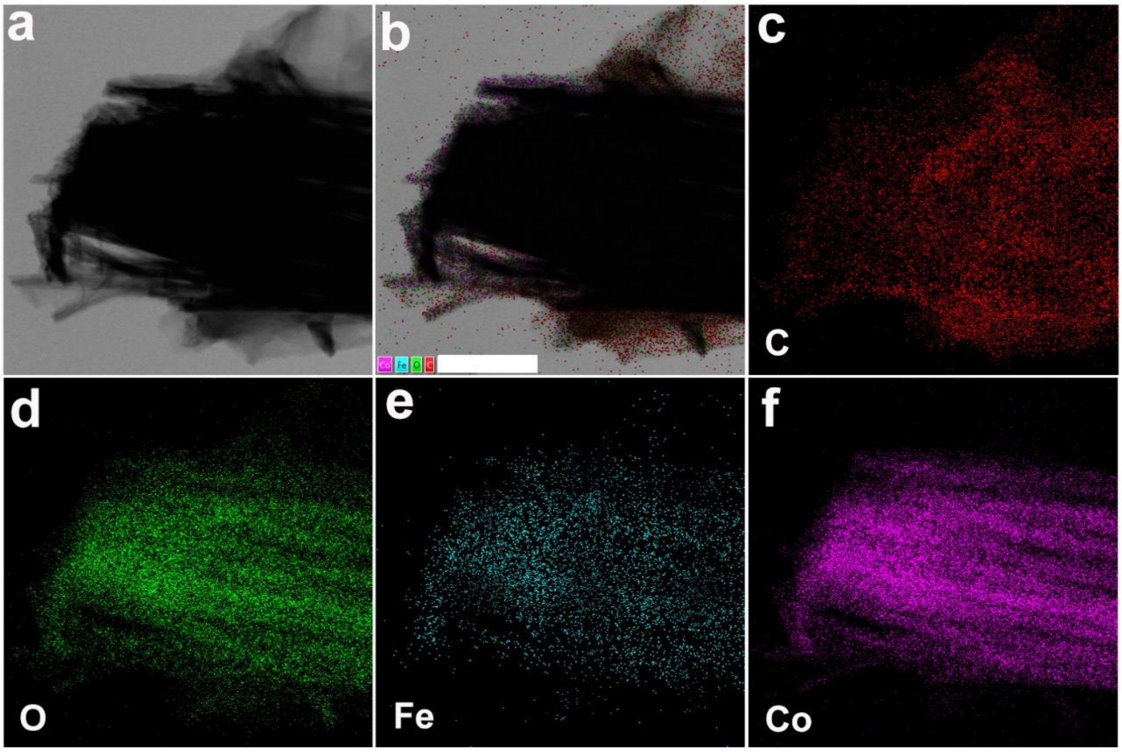

Supplementary Figure 4. Elemental mapping test

a Typical scanning TEM and corresponding elemental mapping images of **b** overlapping, **c** C, **d** O, **e** Fe, and **f** Co atoms in the e-ICLDH@GDY nanosheets. This also indicates the volume expansion behavior of e-ICLDH@GDY. Scale bars: 300 nm.

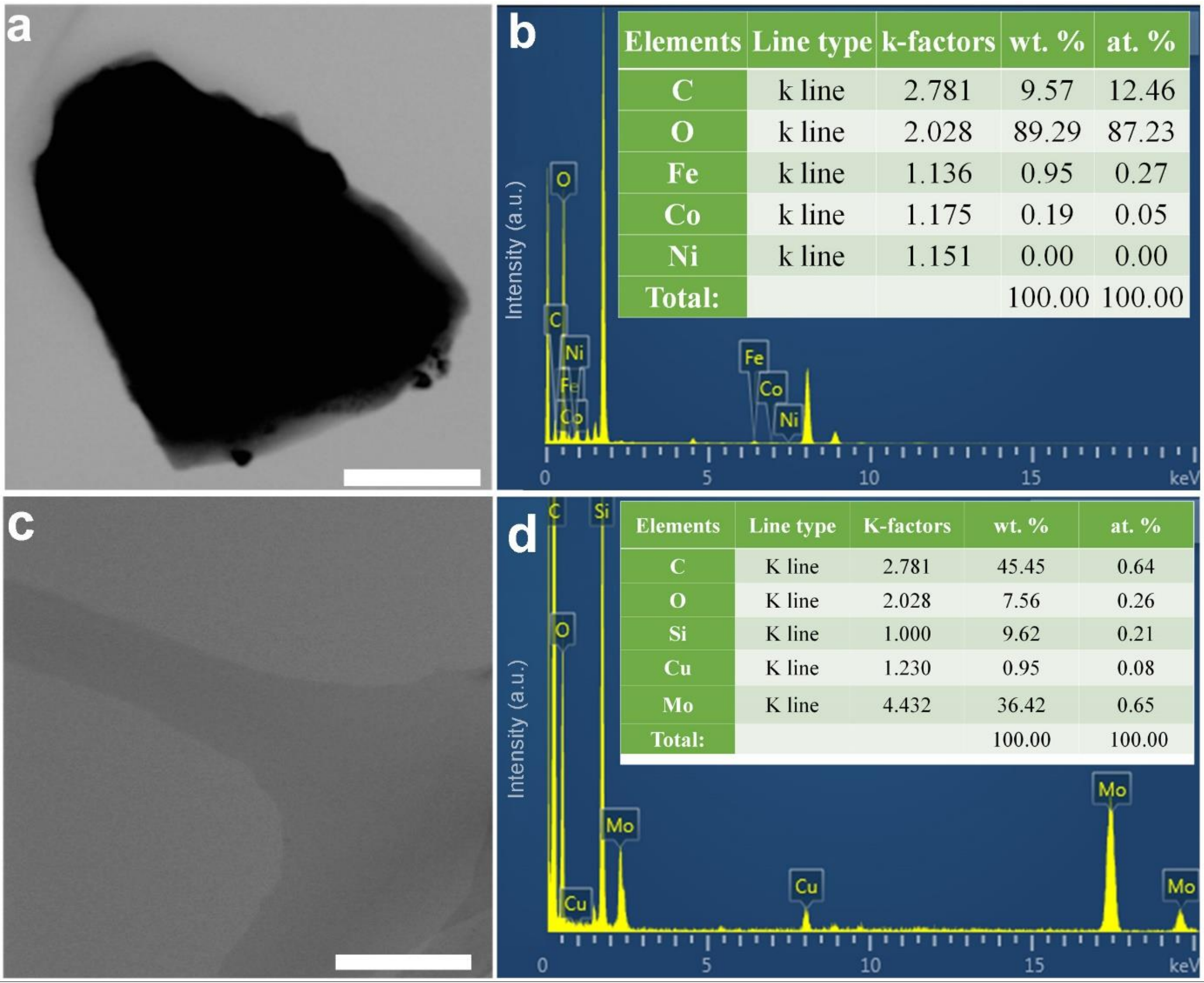

Supplementary Figure 5. Energy dispersive X-ray spectroscopy (EDS) analysis

a STEM image and **b** corresponding EDS pattern of e-ICLDH@GDY nanosheet. Inset of **b**: the relative content of each element. **c** STEM image and **d** corresponding EDS pattern of the pure molybdenum grid. Inset of **d**: the relative content of each element. Scale bars: a, 500 nm; c, 500 nm.

It needs to be mentioned that the EDS results shown in Supplementary Figure 4a and 4b were measured on a molybdenum (Mo) grid. It should be mentioned that the peak at ~8.9 keV corresponding to Cu was ascribed from to the TEM system, for example, the sample rod. In order to clarify this issue, a control measurement on pure molybdenum grid was conducted, as shown in Supplementary Figure 4c and 4d.

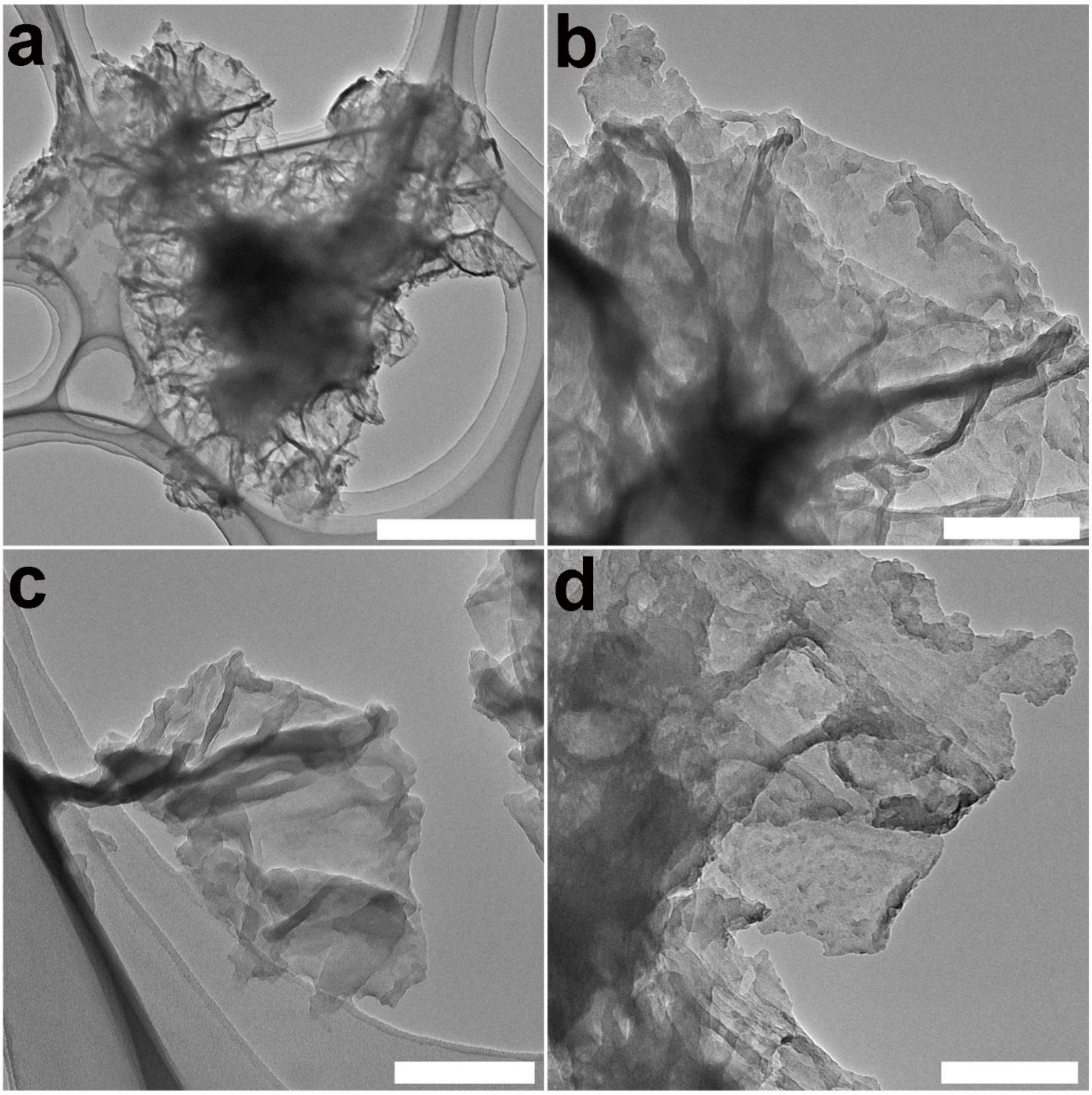

Supplementary Figure 6. TEM characterization of the samples after being treated using concentrated nitric acid

a-d TEM images of GDY nanosheets obtained after the removal of metal species. Scale bars: a, 500 nm; b-d, 100 nm.

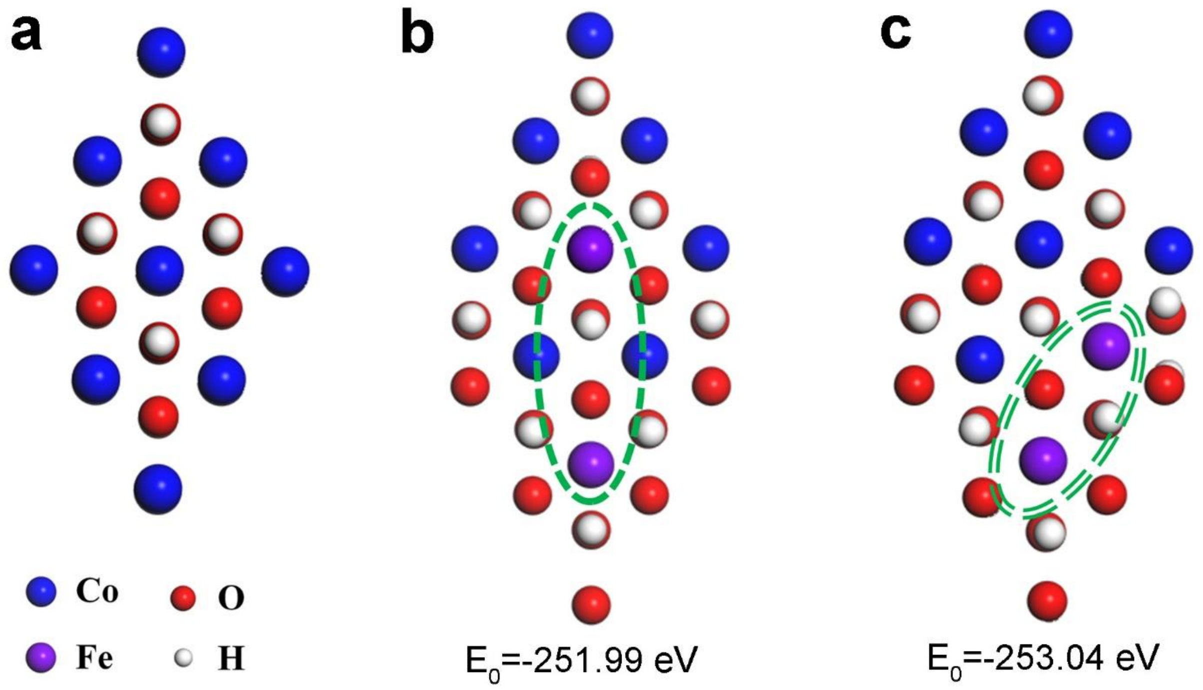

Supplementary Figure 7. Optimization of the structures

a The crystal structure of the 3×3 supercell of CLDH. **b,c** Typical configurations of ICLDH with Fe atoms at different sites of the symmetry systems and corresponding binding energies. The configuration **c** with lower energy demonstrates that it is more stable and thus is chosen as the further investigated model.

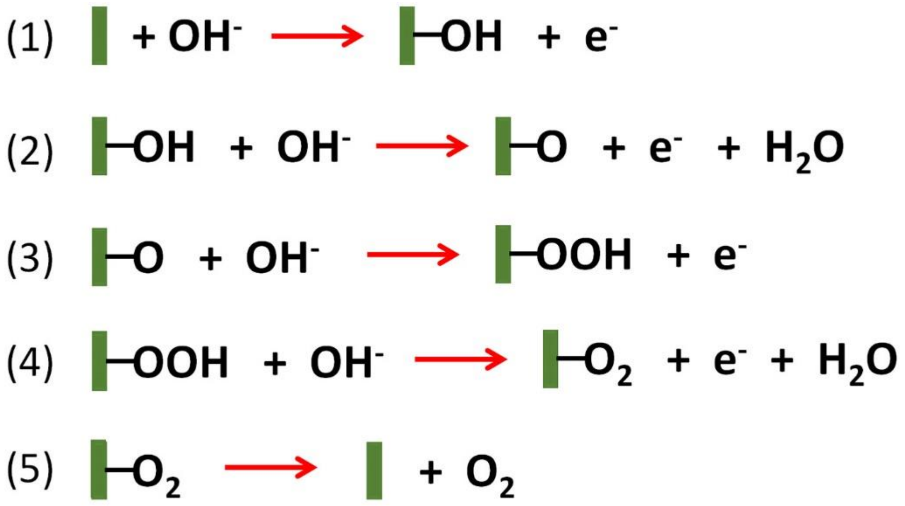

Supplementary Figure 8. Schematic representation of the general mechanism for OER in alkaline solution

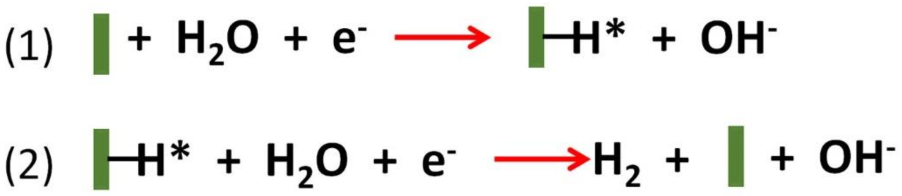

Supplementary Figure 9. Schematic representation of the general mechanism for HER in alkaline solution

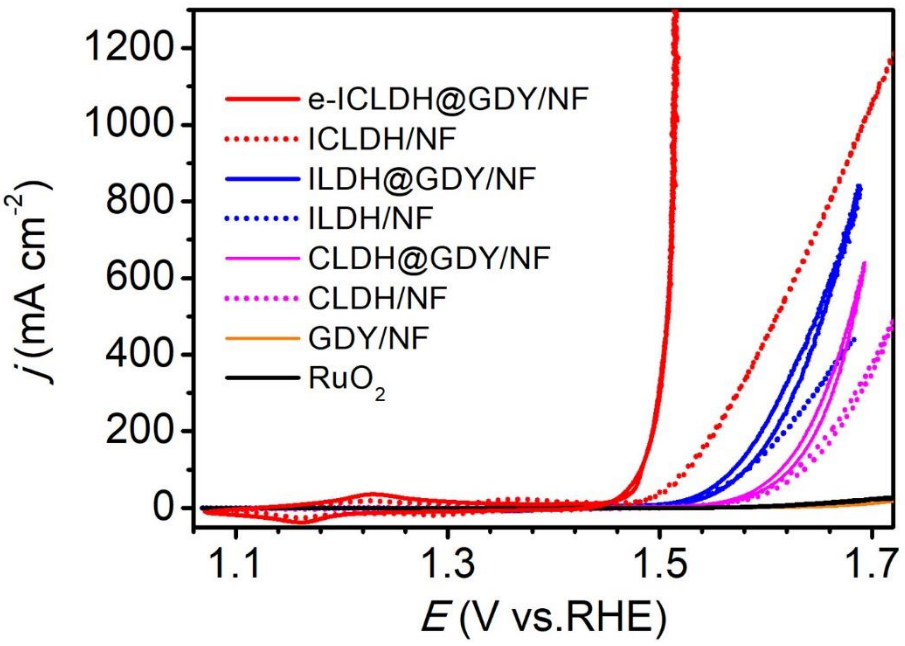

Supplementary Figure 10. OER performances of the as-synthesized samples

OER cyclic voltammetry (CV) curves for e-ICLDH@GDY/NF, ICLDH/NF, ILDH@GDY/NF, ILDH/NF, CLDH@GDY/NF, CLDH/NF, GDY/NF and RuO_2, respectively.

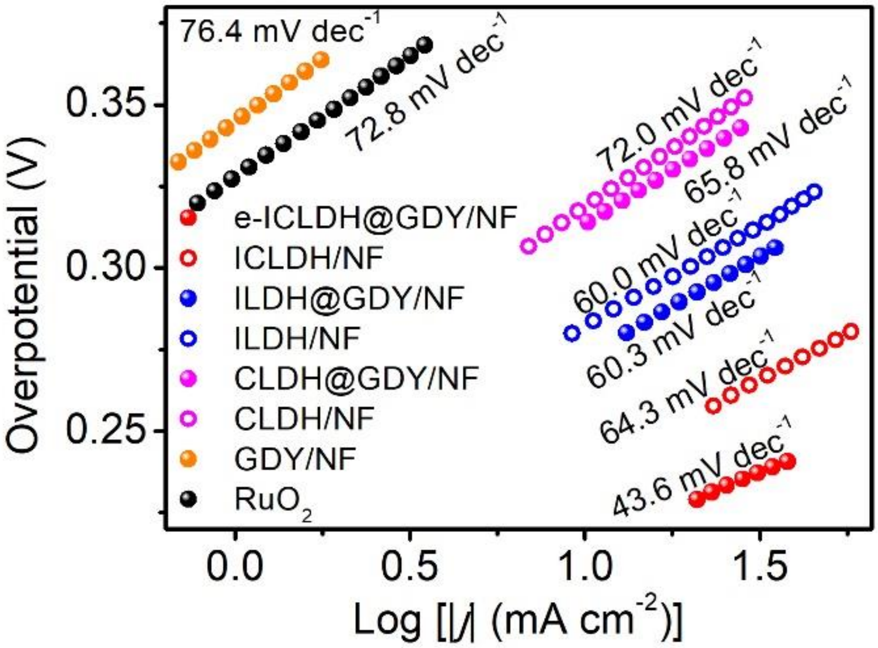

Supplementary Figure 11. Tafel plots together with Tafel slopes of the as-synthesized samples

Tafel plots together with Tafel slopes for e-ICLDH@GDY/NF, ICLDH/NF, ILDH@GDY/NF, ILDH/NF, CLDH@GDY/NF, CLDH/NF, GDY/NF and RuO_2, respectively.

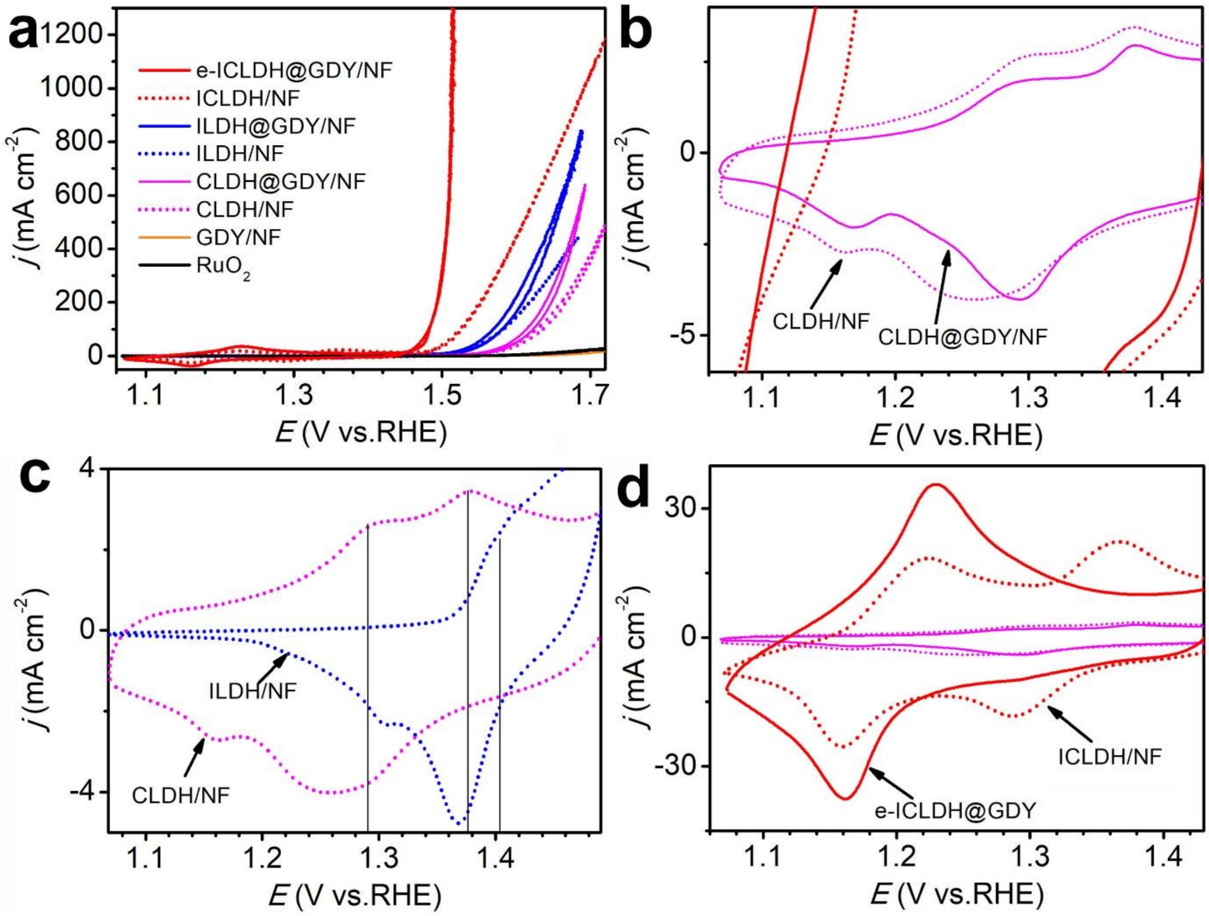

Supplementary Figure 12. Analysis on the obtained LSV curves

a OER cyclic voltammetry (CV) curves for e-ICLDH@GDY/NF, ICLDH/NF, ILDH@GDY/NF, ILDH/NF, CLDH@GDY/NF, CLDH/NF, and GDY/NF, respectively. **b** Magnifications of the pure CLDH/NF and CLDH@GDY/NF curves. **c** Magnifications of the pure CLDH/NF and ILDH/NF curves. **d** Magnifications of the ICLDH/NF and e-ICLDH@GDY/NF curves.

Supplementary Figure 12a shows the OER cyclic voltammetry (CV) curves of as-prepared samples. As shown in Supplementary Fig. 12b, the CV for pure CLDH/NF and CLDH@GDY/NF exhibit two pronounced peaks in the anodic process. The first peak located at around 1.28 V mainly result from the oxidation of Co^{2+} to Co^{3+}, and the latter at 1.36 V corresponds to oxidation of Co^{3+} to Co^{4+} [20–22]. For pure ILDH (Supplementary Fig. 12c), the oxidation peak was observed at round 1.41 V[23]. The CV of e-ICLDH@GDY/NF (Supplementary Fig. 12d) shows only one broad anodic peak at around 1.25 V and a cathodic peak at around 1.16 V. The redox behavior of the e-ICLDH@GDY/NF sample is mainly attributed to the Co^{2+}/Co^{3+} and Co^{3+}/Co^{4+} redox pairs and contribution from iron[21,24-26]. This

has already been reported in alkaline solutions. Such high charge densities would be particularly beneficial for the improvement of the OER catalytic activity[21,24].

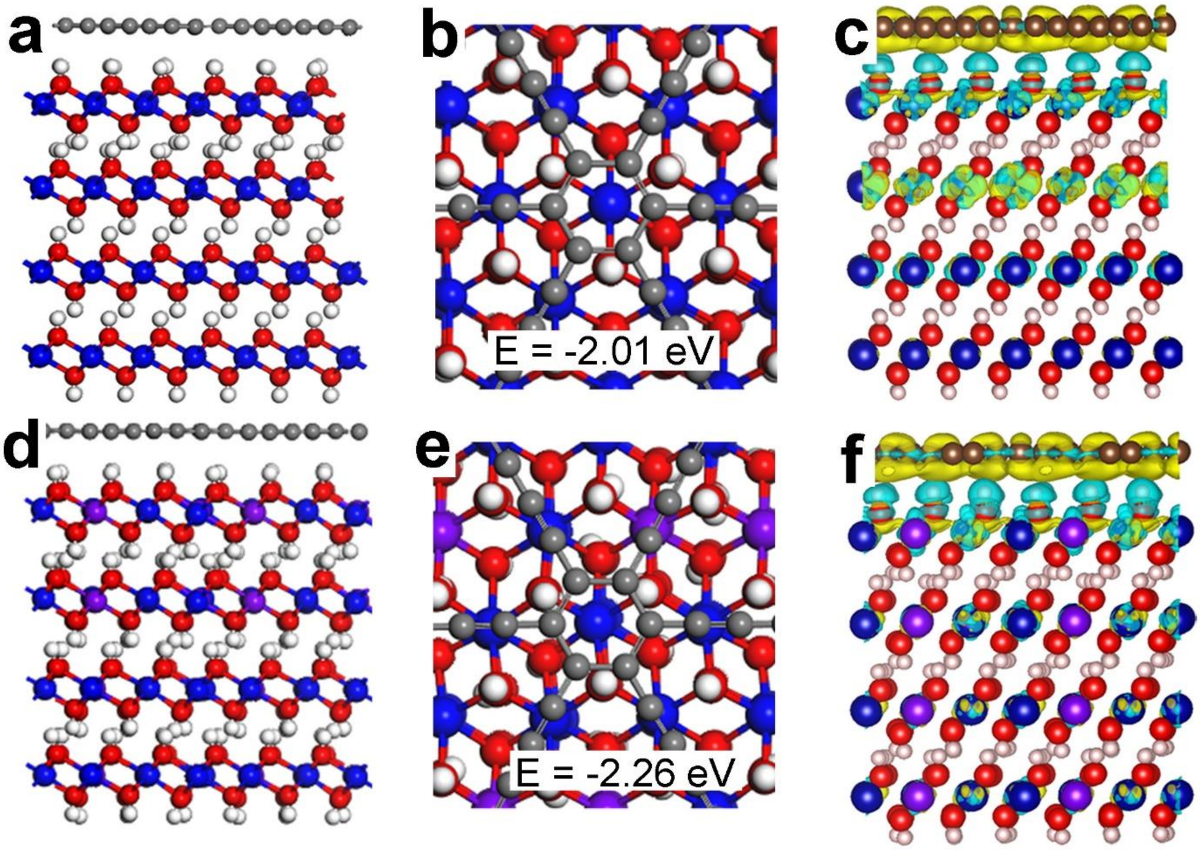

Supplementary Figure 13. Structural optimization

a Side and **b** top views of the stable configuration of CLDH@GDY. **c** Charge density difference for the stable configuration of CLDH@GDY. **d** Side and **e** top views of the stable configuration of ICLDH@GDY. **f** Charge density difference for the stable configuration of ICLDH@GDY. The values of E in **b** and **e** are the binding energies between GDY and the LDHs. Yellow and blue colors in **c** and **f** indicate charge accumulation and loss, respectively.

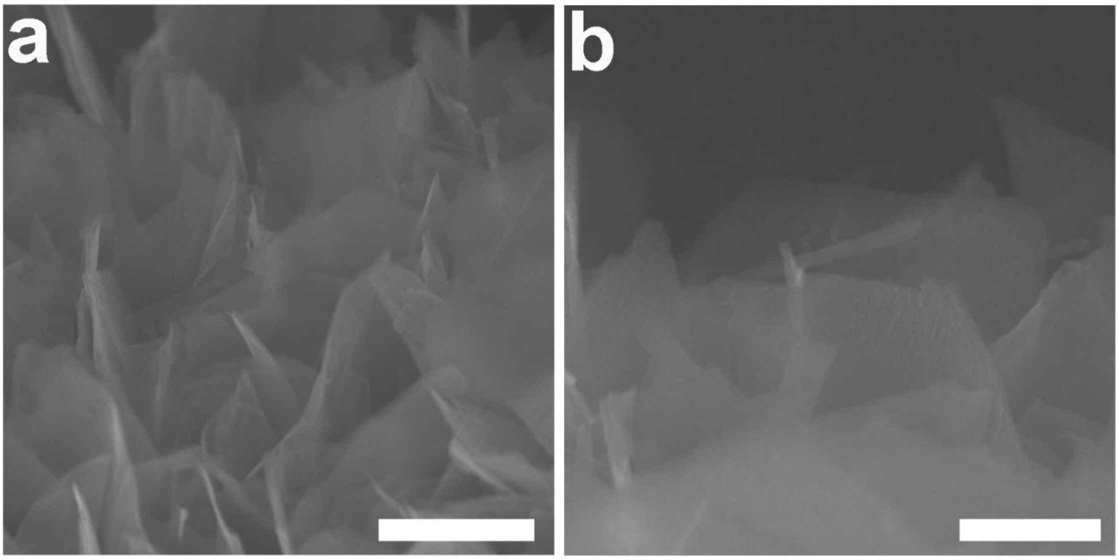

Supplementary Figure 14. Morphological characterizations

a Low- and **b** high-magnification SEM images of e-ICLDH@GDY/NF recorded after OER cycling tests in 1.0 M KOH. Scale bars: a, 1 μm; b, 500 nm.

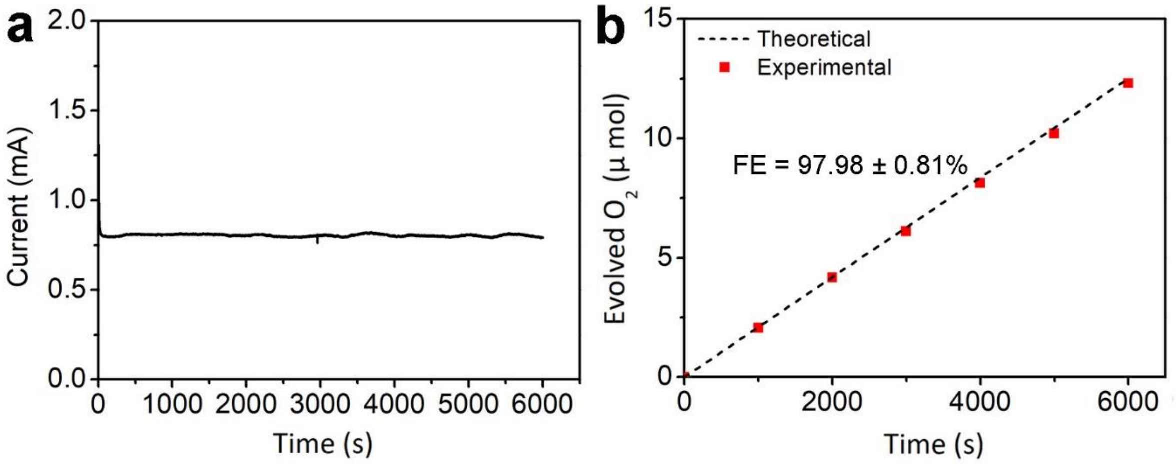

Supplementary Figure 15. Faradaic efficiency test for OER

a Current-time curve and **b** Faradaic efficiency of e-ICLDH@GDY/NF for OER**.**

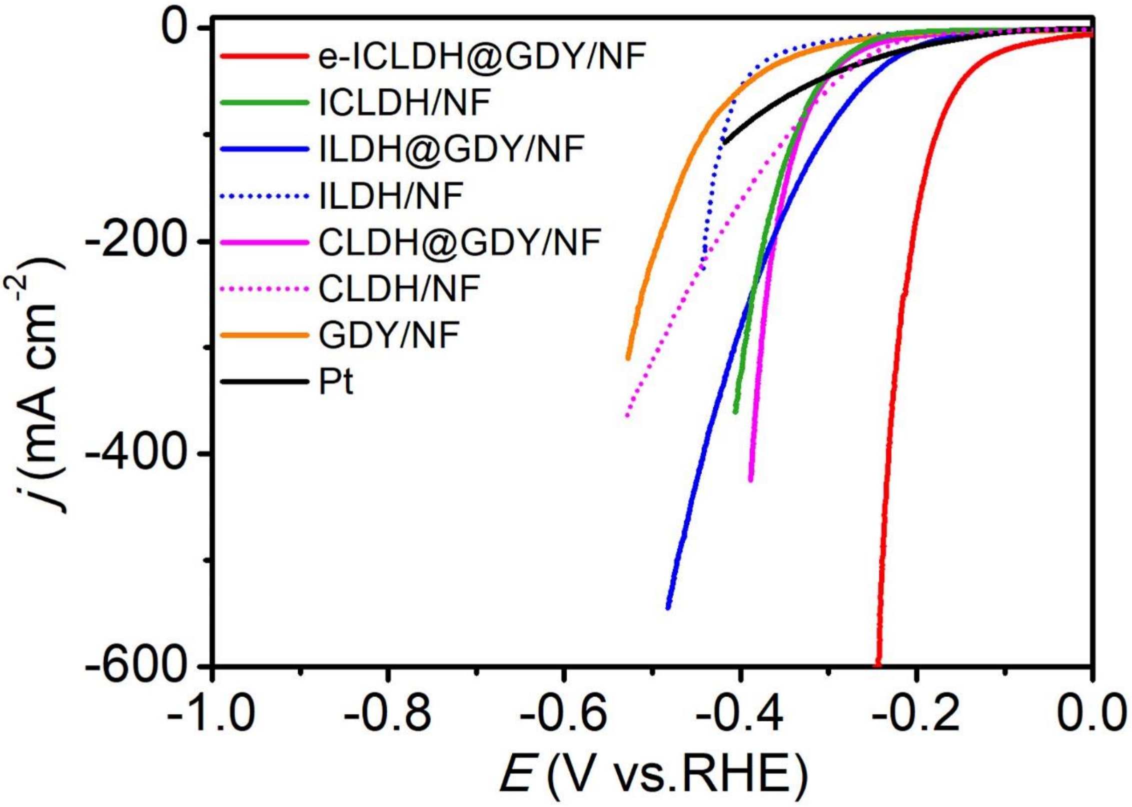

Supplementary Figure 16. HER performances of the as-synthesized samples

HER polarization curves for e-ICLDH@GDY/NF, ICLDH/NF, ILDH@GDY/NF, ILDH/NF, CLDH@GDY/NF, CLDH/NF, GDY/NF and Pt, respectively.

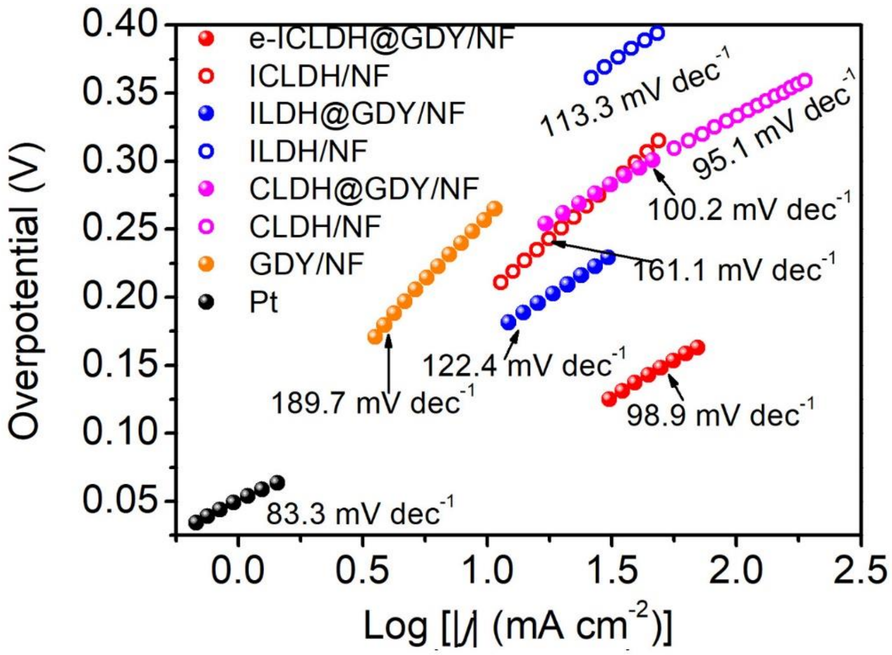

Supplementary Figure 17. Tafel plots together with Tafel slopes for the as-synthesized samples

Tafel plots together with Tafel slopes for e-ICLDH@GDY/NF, ICLDH/NF, ILDH@GDY/NF, ILDH/NF, CLDH@GDY/NF, CLDH/NF, GDY/NF and Pt, respectively, for HER.

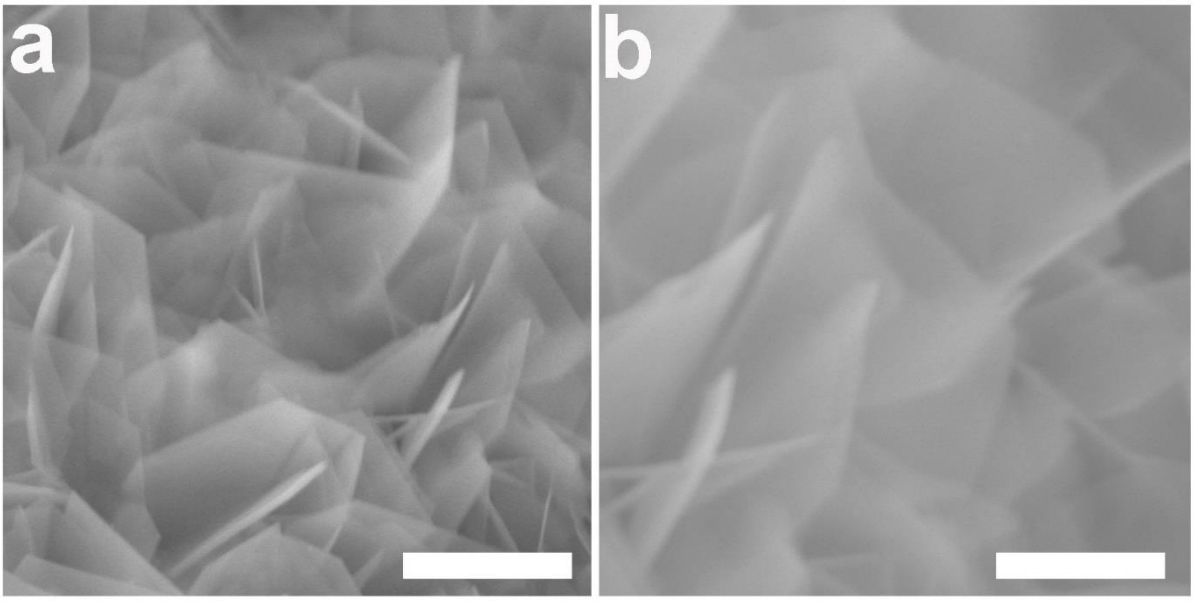

Supplementary Figure 18. Morphological characterizations

a Low- and **b** high-magnification SEM images of e-ICLDH@GDY/NF recorded after HER cycling tests in 1.0 M KOH. Scale bars: a, 1 μ,; b, 500 nm.

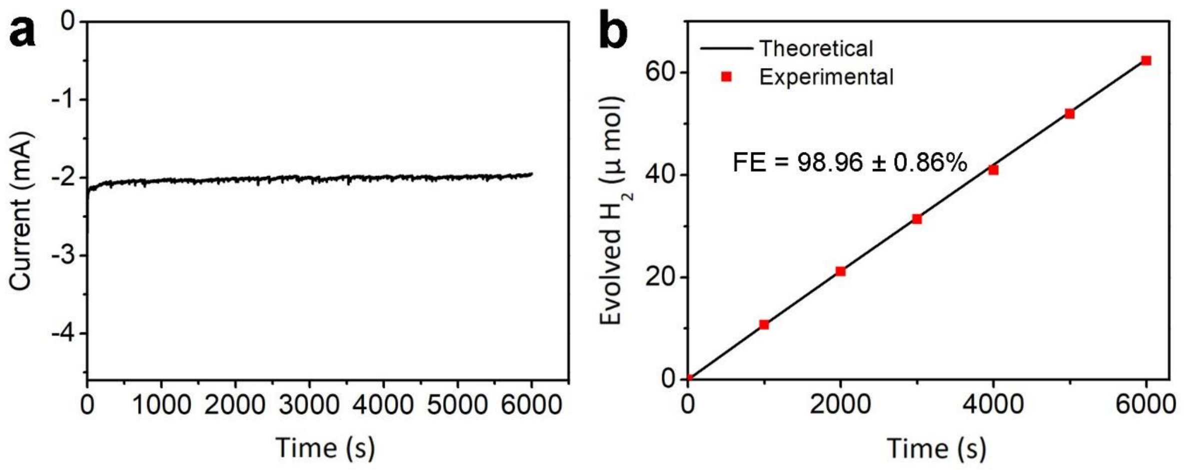

Supplementary Figure 19. Faradaic efficiency test for HER

a Current-time curve and **b** Faradaic efficiency of e-ICLDH@GDY/NF for HER.

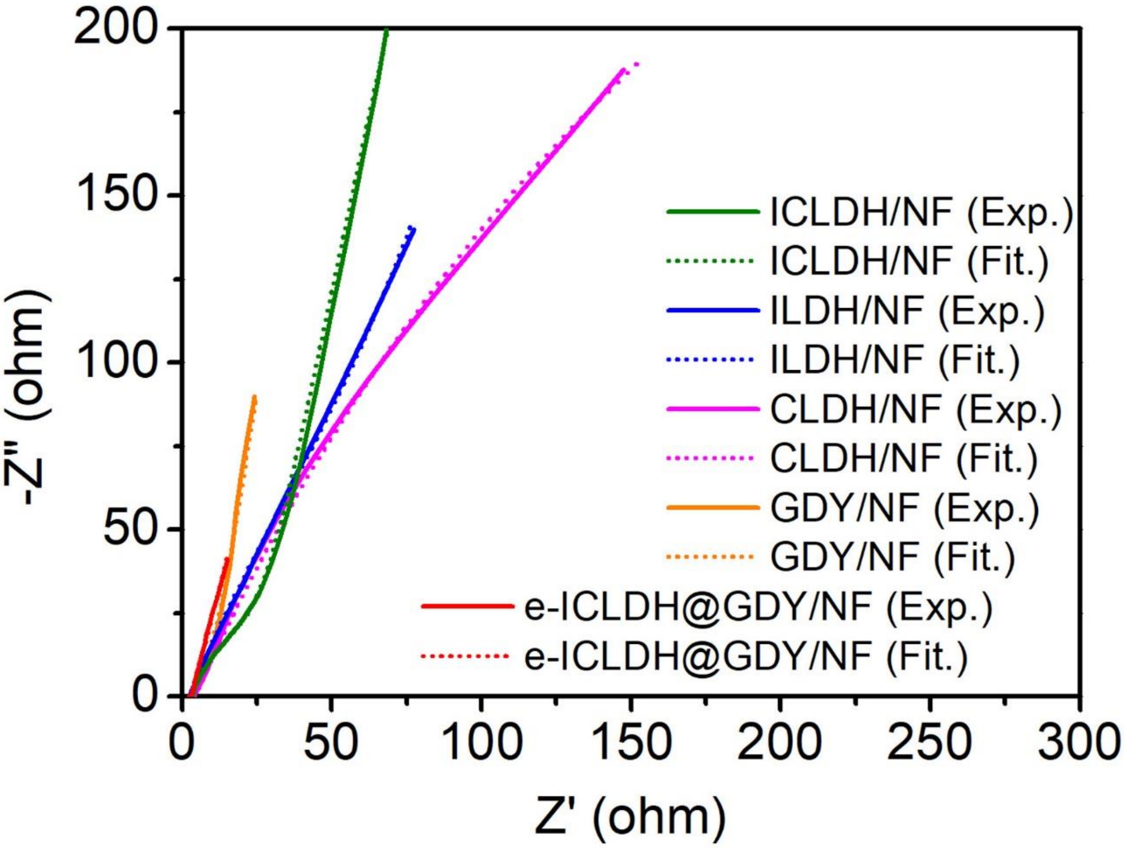

Supplementary Figure 20. Experimental (solid line) and fitted (dashed line) Nyquist plots for as-synthesized samples

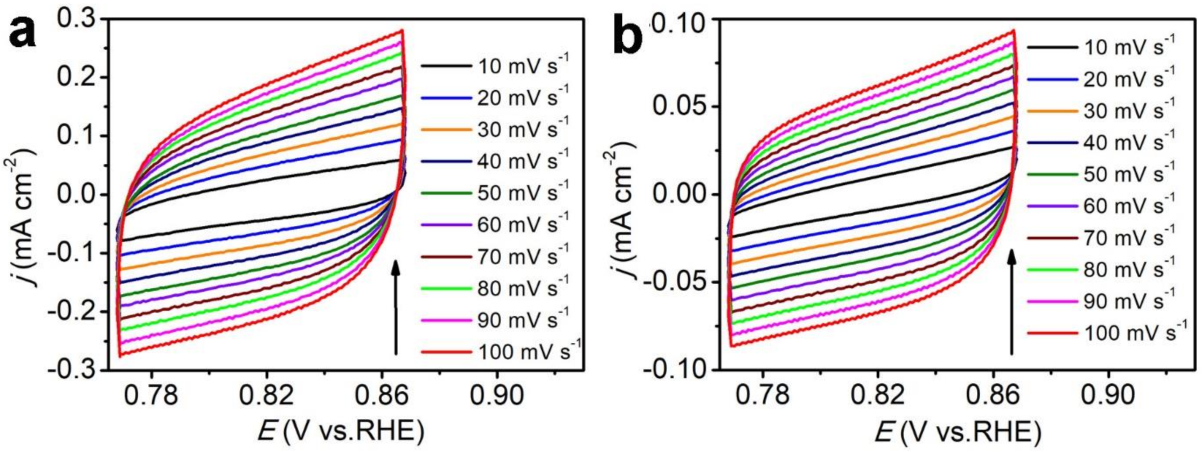

Supplementary Figure 21. CV measurements at different scan rates.

CV curves of **a** e-ICLDH@GDY/NF and **b** pristine ICLDH /NF in the potential range of 0.768–0.868 V vs. RHE at scan rates from 10 mV s^{-1} to 100 mV s^{-1}.

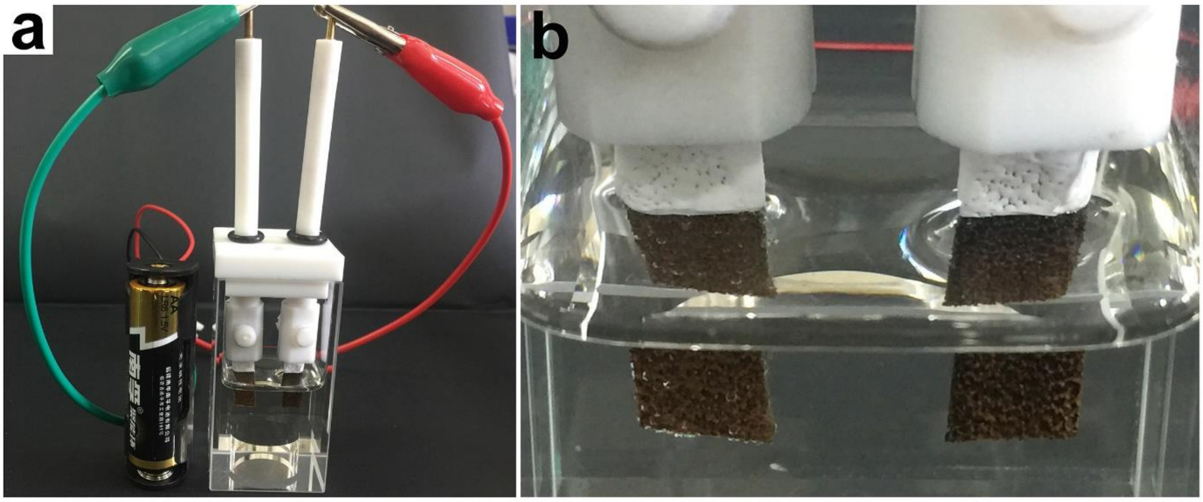

Supplementary Figure 22. Overall water-splitting device

a,b Photograph of a water-splitting device driven by a single AA battery (≈1.5 V).

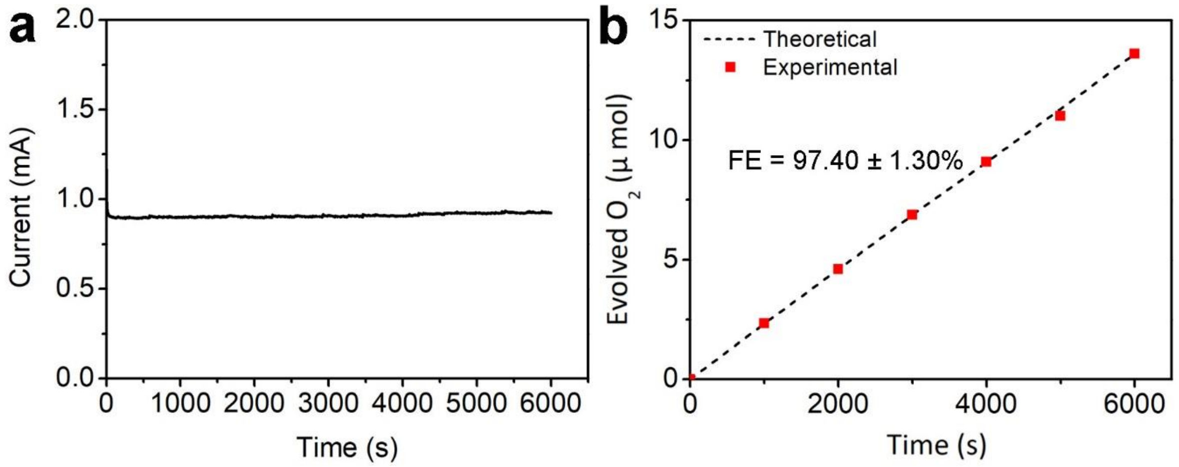

Supplementary Figure 23. Faradaic efficiency for OWS

a Current-time curve and **b** Faradaic efficiency of e-ICLDH@GDY/NF for OWS.

Supplementary Tables

Supplementary Table 1. Comparison of the OER performances of e-ICLDH@GDY/NF with the state-of-the-art electrocatalysts in 1.0 M KOH.

Electrocatalyst	*j* [mA cm^{-2}]	*η* [mV] @ *j*	Tafel slope [mV dec^{-1}]	Ref.
e-ICLDH@GDY/NF	10	216	43.6	*This work*
	50	238		
	100	249		
	500	275		
	1000	278		
IFONFs-45	10	260	55	*Nat. Commun.* **9**, 1809 (2018)
(Ni, Fe)OOH	1000	289		*Energy Environ. Sci.* DOI: 10.1039/C8EE00927A (2018)
FeOOH/Co/FeOOH HNTAs–NF	31.3	250	92.3	*Angew. Chem. Int. Ed.* **55**, 3694 –3698 (2016)
NiFeRu-LDH	10	225		*Adv. Mater.* **30**, 1706279 (2018)
Co_1Mn_1CH/NF	30	294	-	*J. Am. Chem. Soc.* **139**, 8320–8328 (2017)
NiCo@$NiCoO_2$/C PMRAs	20	366	83.97	*Adv. Mater.* **30**, 1705442 (2018)
Fe–CoP/Ti	10	230	67	*Adv. Mater.* **29**, 1602441 (2017)
NF@NC–$CoFe_2O_4$/C NRAs	10	240	45	*Adv. Mater.* **29**, 1604437 (2017)
N-Ni_3S_2/NF 3D	100	330	70	*Adv. Mater.* **29**, 1701584 (2017)
NiFe LDH-NS@DG10	10	210	52	*Adv. Mater.* **29**, 1700017 (2017)
S,N–Fe/N/C–CNT	10	370	82	*Angew. Chem. Int. Ed.* **56**, 610 –614 (2017)
MoS_2	10	235	46	*Nat. Commun.* **8**, 15377 (2017)
Ni_3S_2 nanorods/Ni	10	187	159.3	*Energy Environ. Sci.* **6**, 2921–2924 (2013)

Supplementary Table 2. Comparison of the HER performances of e-ICLDH@GDY/NF with other state-of-the-art electrocatalysts in 1.0 M KOH.

Electrocatalysts	j [mA cm^{-2}]	η [mV] @ j	Tafel slope [mV dec^{-1}]	Ref.
e-ICLDH@GDY/NF	10	43	98.9	*This work*
	50	174		
	100	215		
	500	239		
	1000	256		
NiFe-LDH/NF	10	210	58.9	*Science* **345**, 1593–1596 (2014)
NiFe LDH-NS@DG10	20	115	110	*Adv. Mater.* **29**, 1700017 (2017).
EG/$Co_{0.85}Se$/NiFe-LDH	10	265	160	*Energy Environ. Sci.* **9** 478–483 (2016)
HNDCM-100,000-1,000/Co	10	158	93.4	*Nat. Commun*. **8**, 13592 (2017).
Co_1Mn_1CH/NF	10	180	-	*J. Am. Chem. Soc.* **139**, 8320-8328 (2017).
P-CoO NRs	10	208	164	*Nat. Commun*. **8**, 1509 (2017).
Two-cycle CoO/CNF	10	88	150	*Nat. Commun*. **6**, 7261 (2015).
CoN_x/C	10	170	129	*Nat. Commun*. **6**, 7992 (2015).
Ni3S2/NF	10	223	123.3	*J. Am. Chem. Soc.* **137**, 14023– (2015).
$FeSe_2$	10	176	-	*Angew. Chem. Int.Ed.* ***56***, 10506 –10510 (2017).

Supplementary Table 3. Impedance parameter values derived from the fitting to the equivalent circuit for the impedance spectra recorded in 1.0 M KOH solution.

Samples	R_s [Ω]	Q_1 [Ss^{-n}]	n_1	R_{ct} [Ω]	Q_2 [Ss^{-n}]	n_2	R_{ad} [Ω]
e-ICLDH@GDY/NF	2.46	0.77	0.21	1.42	0.23	0.83	4506
ICLDH/NF	3.45	2.9×10^{-4}	0.83	21.55	4.7×10^{-4}	0.87	1.3×10^{4}
ILDH@GDY/NF	2.47	5.4×10^{-3}	0.69	96.43	4.7×10^{-3}	0.89	2.4×10^{4}
CLDH@GDY/NF	3.41	7.6×10^{-3}	0.71	1042	2.6×10^{-3}	0.80	0.98
GDY/NF	3.78	7.8×10^{-3}	0.65	23.63	1.9×10^{-3}	1.0	1310

Supplementary Table 4. Comparison of the TOF values of our catalysts with recently reported ones.

Electrocatalyst	TOF (S^{-1}) for HER	TOF (S^{-1}) for OER	Ref.
e-ICLDH@GDY/NF	8.44 at 200 mV	2.34 at 250 mV	*This work*
IFONFs-45	0.2770 at 100 mV	0.2141 at 368 mV	*Nat. Commun.* **9**, 1809 (2018)
NiFe/Ni-P	0.33 at 130 mV	0.13 at 250 mV	*Nat. Commun.* **9**, 2014 (2018).
NiFe-MOF	2.8 at 400 mV	3.8 at 400 mV	*Nat. Commun.* **8**, 15341 (2017).
G-Pt_4Ni/GF	2.45 at 60 mV	0.85 (G-Ni_4Fe/GF) at 348 mV	*Adv. Energy Mater.* 1800403 (2018).
Co-Ni_3N	0.1459 at 290 mV	0.0134 at 350 mV	*Adv. Mater.* **30**, 1705516 (2018).
2D-NiSe	0.75 at 468 mV	1.87 at 250 mV	*Adv. Energy Mater.* **8**, 1702704 (2018).

Supplementary Table 5. Comparison of the electrochemical performances of e-ICLDH@GDY/NF for overall water splitting in 1.0 M KOH with recently reported bifunctional electrocatalysts.

Electrocatalyst	*j* [mA cm^{-2}]	Overall voltage [V] @ *j*	Ref.
e-ICLDH@GDY/NF	10	1.43	*This work*
	100	1.46	
	1000	1.49	
NiFe LDH/Ni foam	10	1.70	*Science* **345**, 1593–1596 (2014).
NiFe-NiMo	10	1.51	*Nat. Commun.* **9**, 2014 (2018).
$NiFeO_x$/CFP	10	1.51	*Nat. Commun.* **6**, 7261 (2015).
NiFe LDH	20	1.50	*Nat. Commun.* **5**, 4695 (2014).
Se-(NiCo)S/OH	10	1.60	*Adv. Mater.* **30**, 1705538 (2018).
Co_2P NC	10	1.56	*Adv. Mater.* **30**, 1705796 (2018).
Fe–CoP	10	1.60	*Adv. Mater.* **29**, 1602441 (2017).
NiFe LDH-NS@DG	20	1.50	*Adv. Mater.* **29**, 1700017 (2017)
CoP/NCNHP	10	1.64	*J. Am. Chem. Soc.* **140**, 2610–2618 (2018).
Co_1Mn_1CH/NF	10	1.67	*J. Am. Chem. Soc.* **139**, 8320−8328 (2017).
Co_3O_4 microtube arrays	10	1.63	*Angew. Chem. Int. Ed.* **56**, 1324–1328 (2017).
Hierarchical $NiCo_2O_4$ hollow microcuboids	10	1.65	*Angew. Chem. Int. Ed.* **55**, 6290–6294 (2016).
	20	1.74	
Ni_5P_4	10	1.70	*Angew. Chem. Int. Ed.* **54**, 12361–12365 (2015).
(Ni,Fe)OOH	1000	1.657	*Energy Environ. Sci.* **2018**, DOI: 10.1039/C8EE00927A.
Ni-Co-P hollow nanobricks	10	1.62	*Energy Environ. Sci.* **11**, 872-880 (2018)
np-$Co_{1.04}Fe_{0.96}P$	10	1.53	*Energy Environ. Sci.* **9**,

			2257-2261 (2016)
EG/$Co_{0.85}Se$/NiFe–LDH	10	1.67	*Energy Environ. Sci.* **9**, 478-483 (2016)
	20	1.71	

Supplementary Methods

Calculation details DFT calculations in this work were performed using the VASP program. The exchange-correlation interaction was treated using the PBE functional of GGA. The energy cutoff was set to be 400 eV, and the 3 × 3 × 1 Monkhorst–Pack grid was used to sample the Brillouin zone integration. Spin polarization was considered throughout the calculations. During the geometry optimization, the convergence standards were set as follows: the energy and the force were less than 1.0×10^{-5} eV/atom and 0.01 eV $Å^{-1}$, respectively. To avoid the periodic interactions, a vacuum space as large as 20 Å was used along the c direction. The DFT-D2 force-field approach was employed to consider the van der Waals (vdW) interaction. The electron occupancies were determined according to the Fermi scheme with the energy smearing of 0.1 eV.

The original unit cell of CLDH was obtained from the ICSD database (No.88940). To meet the optimal proportion in experiment and avoid the lattice mismatch between GDY and pure CLDH and ICLDH as far as possible, 1×1 primitive cell of GDY, 3×3 supercell of CLDH and ICLDH, and a proportion of Co/Fe=7:2 were employed. The constructed supercell of CLDH is shown in Supplementary Fig. 3. There are two configurations for Fe doping in different sites of the symmetry systems of I_2C_7LDH (Supplementary Fig. 3). The calculated energies values are -251.99 eV and -253.04 eV, respectively. The configuration 2 with lower energy demonstrates that it is more stable and thus is chosen as the further investigated model.

For electronic structure and energetic pathway studies, we chose rotational invariant PBE+U calculations by CASTEP[1–2], where the Hubbard-U parameters self-consistently determined for the Fe-3d, Co-3d, C-2p and O-2p orbitals by our new linear response method[3–10]. For the ground state structural optimization, the algorithm of Broyden-Fletcher-Goldfarb-Shannon (BFGS) has

been chosen. The plane-wave basis set for expressing the valence electronic states has been used with a kinetic cutoff energy of 750 eV. To guarantee the convergence and avoid spin-charge sloshing effect, the ensemble DFT (EDFT) method of Marzari *et al.*[11] is used for electronic minimization process.

Pseudized CoFe-LDH model has been built with Co:Fe ratio of 7:2 and four layered thick. The single GDY layer model was initially interfaced with 4-layered LDH structure. The vacuum thickness is set to 10 Å. To balance the computational loading, the Monkhost-Pack reciprocal k-point sampling was performed using Gamma-center-off special k-points[12] with convergence test guided. Total energy for each step is converged to less than 1.0×10^{-6} eV per atom. The Hellmann-Feynman forces on the atom were converged to reach less than 0.001 eV/Å.

The Fe, Co, C, O, and H norm-conserving pseudopotentials are generated using the OPIUM code in the Kleinman-Bylander projector form[13]. We chose the (3*d*, 4*s*, 4*p*), (2*s*, 2*p*), and (1*s*) states as the valence states of Fe, Co, C, O, and H atoms respectively. The RRKJ method is chosen for the optimization of the pseudopotentials[14]. The Hubbard U parameters on the Fe-3d orbitals is self-consistently to be U_d=2.01 eV, U_d=3.51 eV for Co-4d, and U_p=2.85 eV for O-2p, respectively.

Calculation of TOF According to the previously reported method[15-18], we carried out similar calculation method. As the exact number and nature of hydrogen binding sites is not known, we estimated the number of active sites as the as possible active sites from the roughness factor (eq. 1).

$$\frac{\#\, Surface\ sites}{cm^2\ geometric\ area} = \frac{\#\, Surface\ sites\ (flat\ standard)}{cm^2\ geometric\ area} \times Roughness\ factor \qquad (1)$$

The roughness factor (R_f) can be determined by the electrochemically double-layer capacitance (C_{dl}). The specific capacitance can be converted into an electrochemical active surface area

(ECSA) using the specific capacitance value for a flat standard with 1 cm^2 of real surface area. According to previous reports[16,19], we assume 60 μF cm^{-2} for a flat electrode and the surface sites of 2 $\times 10^{15}$ for the flat standard electrode. As a result, the number of surface active sites for e-ICLDH@GDY is calculated to be 0.067 $\times 10^{18}$ surface sites/cm^2.

The TOF values can then be obtained according to the following formulas:

$$TOF = \frac{\#\,Total\ Hydrogen\ Turn\ Overs/cm^2 geometric\ area}{\#\,Surface\ Sites\ /cm^2\ geometric\ area} \quad (2)$$

The number of total hydrogen turn overs is calculated from the current density extracted from the LSV curves:

$$\#\,Total\ Hydrogen\ Turn\ Overs$$

$$= \left(j\frac{mA}{cm^2}\right)\left(\frac{1C\ s^{-1}}{1000\ mA}\right)\left(\frac{1\ mol\ e^-}{96485.3\ C}\right)\left(\frac{1\ mol\ H_2}{2\ mol\ e^-}\right)\left(\frac{6.022\ \times 10^{23}\ H_2\ molecules}{1\ mol\ H_2}\right)$$

$$= 3.12\ \times 10^{15}\ \frac{H_2/s}{cm^2}\ per\ \frac{mA}{cm^2} \quad (3)$$

TOF per site for e-ICLDH@GDY at different overpotentials (η) is calculated as follows:

$$TOF = \frac{3.12\ \times\ 10^{15}}{0.067 \times 10^{18}} \times j\ = 0.047\ \times j \quad (4)$$

j corresponds to the current density at different overpotentials.

The C_{dl} of pure ICLDH is 0.6 mF cm^{-2}. According to Eq. (1), the number of surface active sites for pure ICLDH is calculated to be 0.02 $\times 10^{18}$ surface sites/cm^2. Thus, TOF per site for e-ICLDH@GDY at different overpotentials (η) can be calculated as follows:

$$TOF = \frac{3.12\ \times\ 10^{15}}{0.02 \times 10^{18}} \times j\ = 0.156\ \times j \quad (5)$$

For HER, at the overpotentials of 50, 100, and 200 mV, the current densities for e-ICLDH@GDY are 11.01, 20.69, and 179.16 mA cm^{-2} respectively. For pure ICLDH, The j at η = 50, 100, and 200 mV are 1.40, 1.58, and 3.55 mA cm^{-2}, respectively. As summarized below,

the e-ICLDH@GDY exhibits much bigger TOF value than the pure ICLDH, suggesting its higher HER catalytic activity.

	Catalysts	η = 50 mV	η = 100 mV	η = 200 mV
**HER**	**e-ICLDH@GDY**	**0.517 s^{-1}**	**0.944 s^{-1}**	**8.44 s^{-1}**
	**ICLDH**	**0.218 s^{-1}**	**0.246 s^{-1}**	**0.554 s^{-1}**

By using the same calculation method, the TOF values of OER was obtained. At η = 250 mV, the TOF values for e-ICLDH@GDY and pure ICLDH are 2.34 and 1.29 s^{-1}, respectively. This result indicates the better OER catalytic activity of e-ICLDH@GDY than pure ICLDH.

Supplementary References:

1. Vladimir, I. A., Aryasetiawan, F. & Lichtenstein, A. I. First-principles calculations of the electronic structure and spectra of strongly correlated systems: the LDA + U method. *J. Phys. Condens. Matter* **9**, 767 (1997).
2. Clark, S. J. *et al.* First principles methods using CASTEP. *zkri* **220**, 567 (2005).
3. Huang, B., Gillen, R. & Robertson, J. Study of CeO_2 and its native defects by density functional theory with repulsive potential. *J Phys. Chem. C* **118**, 24248–24256 (2014).
4. Huang, B. Superiority of DFT+U with non-linear core correction for open-shell binary rare-earth metal oxides: a case study of native point defects in cerium oxides. *Philosophical Magazine* **94**, 3052–3071 (2014).
5. Huang, B. 4f fine-structure levels as the dominant error in the electronic structures of binary lanthanide oxides. *J Comput. Chem.* **37**, 825–835 (2016).
6. Huang, B. Intrinsic deep hole trap levels in Cu_2O with self-consistent repulsive Coulomb energy. *Solid State Commun.* **230**, 49–53 (2016).
7. Huang, B. Strong compensation hinders the p-type doping of ZnO: a glance over surface defect levels. *Solid State Commun.* **237–238**, 34–37 (2016).
8. Huang, B. Unraveling energy conversion modeling in the intrinsic persistent upconverted luminescence of solids: a study of native point defects in antiferromagnetic Er_2O_3. *Phys. Chem. Chem. Phys.* **18**, 13564–13582 (2016).
9. Huang, B. The screened pseudo-charge repulsive potential in perturbed orbitals for band calculations by DFT+U. *Phys. Chem. Chem. Phys.* **19**, 8008–8025 (2017).
10. Hu, J. *et al.* Engineering stepped edge surface structures of MoS_2 sheet stacks to accelerate the hydrogen evolution reaction. *Energy Environ. Sci.* **10**, 593–603 (2017).

11. Marzari, N., Vanderbilt, D. & Payne, M. C. Ensemble density-functional theory for Ab initio molecular dynamics of metals and finite-temperature insulators. *Phys. Rev. Lett.* **79**, 1337–1340 (1997).

12. Probert, M. I. J. & Payne, M. C. Improving the convergence of defect calculations in supercells: An ab initio study of the neutral silicon vacancy. *Phys Rev B* **67**, 075204 (2003).

13. Kleinman, L. & Bylander, D. M. Efficacious form for model pseudopotentials. *Phys. Rev. Lett.* **48**, 1425–1428 (1982).

14. Rappe, A. M., Rabe, K. M., Kaxiras, E. & Joannopoulos, J. D. Optimized pseudopotentials. *Phys. Rev. B* **41**, 1227–1230 (1990).

15. Zhang, J. *et al.* Efficient hydrogen production on MoNi4 electrocatalysts with fast water dissociation kinetics. *Nat. Commun.* **8**, 15437 (2017).

16. Yu, L. *et al.* Ternary $Ni_{2(1-x)}Mo_{2x}P$ nanowire arrays toward efficient and stable hydrogen evolution electrocatalysis under large-current-density. *Nano Energy* **53**, 492–500 (2018).

17. Zhou, H. *et al.* Efficient hydrogen evolution by ternary molybdenum sulfoselenide particles on self-standing porous nickel diselenide foam. Nat. Commun. **7**, 12765 (2016).

18. Kibsgaard, J. & Jaramillo, T.F. Molybdenum phosphosulfide: an active, acid-stable, earth-abundant catalyst for the hydrogen evolution reaction. *Angew. Chem. Int. Ed.* **53**, 14433–14437 (2014).

19. Kibsgaard, J. *et al.* Designing an improved transition metal phosphide catalyst for hydrogen evolution using experimental and theoretical trends. Energy Environ. Sci. 8, 3022–3029 (2015).

20. Chen, Z., Kronawitter, C. X. & Koel, B. E. Facet-dependent activity and stability of Co_3O_4 nanocrystals towards the oxygen evolution reaction. *Phys. Chem. Chem. Phys.* **17**, 29387−29393 (2015).

21. Zhu, C. *et al.* Nickel cobalt oxide hollow nanosponges as advanced electrocatalysts for the oxygen evolution reaction. *Chem. Commun.* **51**, 7851−7854 (2015).

22. Dong, X. *et al.* 3D graphene–cobalt oxide electrode for high-performance supercapacitor and enzymeless glucose detection. *ACS Nano* **6**, 3206–3213 (2012).

23. Zhang, K., Hu, Z., Liu, X., Tao, Z. & Chen J. $FeSe_2$ microspheres as a high-performance anode material for Na-ion batteries. *Adv. Mater.* **27**, 3305–3309 (2015).

24. Yeo, B. S. & Bell, A. T. Enhanced activity of gold-supported cobalt oxide for the electrochemical evolution of oxygen. *J. Am. Chem. Soc.* **133**, 5587–5593 (2011).

25. Meher, S. K. & Rao, G. R. Ultralayered Co_3O_4 for high-performance supercapacitor applications. *J. Phys. Chem. C* **115**, 15646–15654 (2011).

26. Pendashteh, A., Palma, J., Anderson, M. & Marcilla, R. Nanostructured porous wires of iron cobaltite: novel positive electrode for high-performance hybrid energy storage devices. J. *Mater. Chem. A* **3**, 16849–16859 (2015).
