# Supplementary material for: Overall water splitting by graphdiyne-exfoliated and -sandwiched layered double-hydroxide nanosheet arrays
Source: Nat Commun. 2018 Dec 14;9:5309. doi: 10.1038/s41467-018-07790-x (PMC6294247; doi:10.1038/s41467-018-07790-x)
Supplement: Supplementary file 3 — Description of Additional Supplementary Information [file 41467_2018_7790_MOESM3_ESM.pdf]

Description of Additional Supplementary Files

File Name: Supplementary Movie 1
Description: the overall water splitting process driven by a single-cell AA battery with a nominal voltage of 1.5 V. taken from a distance.

File Name: Supplementary Movie 2
Description: the overall water splitting process driven by a single-cell AA battery with a nominal voltage of 1.5 V. taken at a close range.
